# Potential impact of marine heatwaves on the survival and transcriptomic signature of free-living third-stage larvae (L3) of *Anisakis simplex* (Nematoda: Anisakidae)

**DOI:** 10.3389/fvets.2026.1758357

**Published:** 2026-02-06

**Authors:** Raquel Ríos-Castro, Elvira Abollo, Santiago Pascual

**Affiliations:** Ecology and Marine Biodiversity (Ecobiomar) Group, Institute of Marine Research, Spanish National Research Council (IIM-CSIC), Vigo, Spain

**Keywords:** *Anisakis simplex* third stage, cuticle, marine heatwaves, stress, survival, temperature, transcriptomic

## Abstract

**Introduction:**

This experimental study aims investigates the gene expression plasticity of free-living infective third-stage larvae (L3) of Anisakis simplex in response to extreme thermal stress, potentially linked to the increasing occurrence of climate change-induced marine heatwaves.

**Methods:**

Using an RNA-seq approach, the molecular transcriptomic responses of L3 larvae were analyzed under both normal (15 °C) and extreme (28 °C) heating aquarium conditions environmental stress. Data were collected from90 viable L3 in good condition, sampled from viscera of fresh European hake (*Merluccius merluccius*), and subjected to a seawater survival assay simulating the most extreme ocean warming event currently recorded whitin the Spanish Marine Demarcation. A bioinformatic pipeline involving read alignment (STAR), differential expression (DESeq2), and gene ontology enrichment was applied.

**Results:**

The analysis revealed that larvae remained viable for up to 60 days at 15 °C but only a week at 28 °C. Transcriptomic data showed from 241 to 244 DEGs depending on each comparison, with a progressive downregulation of cuticle-related genes, a strong upregulation of peptidases, and coordinated neural modulation; with reduced ion channel activity and enhanced expression of key neurotransmission-related genes. Additionally, there was strong upregulation of genes involved in glycogen degradation and galactose metabolism, along with consistent downregulation of glycolysis-related genes.

**Discussion:**

The transcriptomic patterns describe above indicate that short-term marine heatwave conditions (28 °C) severely compromise viability of free L3 of A. simplex, which clearly prioritize survival processes while suppressing developmental pathways. These findings suggest that the more intense, longer-lasting, and widespread marine heatwaves observed along the Spanish marine demarcation over the last 15 years may influence parasite persistence and transmission of infective stages to paratenic fish host. This highlights the One Health significance of such adaptative response, particularly regarding parasite biogeographical dispersion and associated zoonotic risk.

## 1 Introduction

The life cycle of *Anisakis* Dujardin, 1845 (Rhabditida, Anisakidae) involves several hosts at different trophic levels. The adult stage parasitizes the digestive tracts of cetaceans as final hosts (1). After copulation and oviposition, eggs released by gravid females pass with host feces into the marine environment, where free-living third-stage larvae (L3) hatch from the eggs (2). Several species of mesozooplankton feed on these L3, serving as intermediate hosts (3). After ingestion of infected crustaceans, numerous fish and cephalopod species may serve as poikilotherm paratenic hosts, where the L3 remain in a dormant state until they found the homeotherm final host (4). A marked feeding habit of cetaceans (5) on infected paratenic hosts assures the acquisition of infective L3, and after two molting phases (from L3 to adult) the life cycle is completed. An anthropogenically-induced enlargement of the parasite life cycle contributing to the persistence and dissemination of *Anisakis* spp. [namely “the anthropogenic shortcut,” Cipriani et al. (6)] is the disposal of parasitized viscera into the sea during gutting operations carried out by the fishing fleet. This widespread action in fisheries industries, reintroduces millions of free-L3 of *Anisakis* spp. into the marine food web (7), facilitating their ingestion by intermediate or definitive hosts, and thereby enhancing opportunities for transmission.

Since the success of *Anisakis* life cycle depends on the above host-parasite assemblages but also on the interactions with the surrounding water masses, climate change impacts may further complicate a predictive understanding of the life cycle. Significant long-term warming trend in sea surface temperature (SST) have been detected in Atlantic Ocean in the last years (8–10) with sustained short-term periods characterized by abnormally warm ocean temperatures, particularly in areas of the Spanish Marine Demarcation (11). These discrete yet persistent events of anomalously warm ocean temperatures or marine heatwaves, have the potential to origin severe damage to regional marine ecosystems and reshape species distribution and host-parasite dynamics (9). Remarkably, temperature is one of the factors that influence the rate of biological functions and adaptation of marine parasites (12), mostly in those parasites with life cycles involving both homeothermic and poikilothermic hosts, such as *Anisakis* spp. (13). For example, the hatching time and the survival of hatched larvae of *Anisakis simplex* was inversely related to temperature (5–21 °C) (2), which indicates a preference for colder waters (14). In fact, no records for this species were found in the Mediterranean Sea, with some exceptions from those of the Alboran Sea that maintains similar oceanographic conditions that in the Atlantic waters (15). In contrast, *Anisakis pegreffii*, a species historically associated with the Mediterranean Sea, has been increasingly reported over the last 25 years in temperate Atlantic waters along Iberian Peninsula waters, confirming this area as a hot-hybrid zone for the two sibling species (16–18).

Recent metabolomic and transcriptomic studies indicate that *Anisakis* spp. larvae can modulate their gene expression and metabolite profiles depending on their hosts conditions, reflecting a strategy of host adaptation to survive (4, 13, 19–21). However, less is known about their physiological responses when L3 larvae present in the viscera of ectothermic host are released into the marine environment, for instance following the discard of host viscera. We investigated using high-throughput RNA sequencing the differential gene expression in *A. simplex* L3 exposed to two contrasting temperatures: current ocean temperature and a heat anomaly currently associated with marine heatwaves. The aim of this study was to elucidate the parasite thermal response in terms of survival and molecular adaptation, and to assess whether these responses influence the parasite's survival and its ontogenetic competence.

## 2 Materials and methods

### 2.1 Sampling and survival experiment

Third-stage larvae (L3) of *A. simplex* were carefully removed from the viscera of fresh European hake (*Merluccius merluccius*) sourced from ICES Division ICES VIIj, ensuring minimal mechanical damage. The selection of the sampling area was based on recent genetic–population data demonstrating that only *A. simplex* is present in this area, with no evidence of coexistence with other species of the genus *Anisakis* (18, 22). The larvae were then subjected to a survival experiment in aquaria performed at 15 °C, corresponding to the mean sea surface temperature (SST) of NE Atlantic at the time of the experiment and at 28 °C, representing an extreme surface thermal scenario associated with marine heatwave events that have already been recorded along the Atlantic coast of the Iberian Peninsula in recent years (11). Although adult *M. merluccius* typically inhabit deeper and colder waters, these temperatures reflect conditions in the upper water column, which are relevant for biological material transiting through the water column following fishing activities. During the experiment, L3 *Anisakis* larvae were maintained at both temperatures in aquaria containing seawater. Survival was monitored daily until larval mortality was confirmed, defined by the complete absence of parasite motility and further validated through their fluorescent examination in an UV chamber at 365 nm. Dead larvae were removed from the experimental setup daily. Throughout the experiment, temperature and pH were daily recorded using a datalogger to ensure maintenance of the predefined environmental conditions. At the beginning of the experiment, L3 larvae of *A. simplex* were allocated to two temperature treatments. At each sampling time point, only viable larvae were randomly selected for gene expression analyses. At 15 °C, a total of 90 larvae were sampled across six time points (days 0, 3, 7, 15, 30, and 60), with three biological replicates per time point, each one consisting of a pool of five larvae. At 28 °C, 45 larvae were sampled at days 0, 3, and 7 following the same strategy. Due to the high larval mortality at 28 °C, the experiment at this temperature was stopped after day 7. The collected larvae were immediately preserved in liquid nitrogen until RNA extraction to maintain RNA integrity. Pooling was necessary because individual *Anisakis* L3 larvae do not yield sufficient RNA quantity or quality to enable reliable RNA-Seq library preparation and transcriptome analysis.

### 2.2 RNA isolation and sequencing

Total RNA was extracted directly from pooled samples using mechanical homogenization using a TissueLyser II (Qiagen), followed by NZYol reagent (NZYTech) extraction using manufacturer's instructions. RNA integrity and concentration were assessed using an Agilent 2100 Bioanalyzer. Libraries were prepared with the NEBNext^®^ Ultra™ II Directional RNA Library Prep Kit incorporating poly-A mRNA enrichment, reverse transcription into cDNA, adapter ligation, and quality assessment using the Agilent RNA 6000 Nano Kit and High Sensitivity DNA Kit (Agilent Technologies). Libraries were quantified with a Qubit dsDNA HS Assay Kit (Thermo Fisher Scientific), equimolarly pooled, and purified using Mag-Bind RXNPure Plus beads (Omega Bio-tek). Paired-end sequencing was conducted on an Illumina NovaSeq PE150, generating a total output of 320 gigabases. The resulting reads were deposited in the Sequence Read Archive (SRA) http://www.ncbi.nlm.nih.gov/sra (accessed on 15 May 2025) under BioProject accession PRJNA1371940.

### 2.3 Bioinformatic analysis

For each sample, the quality of paired-end raw reads (R1 and R2) was evaluated using FastQC v.0.12.1 (23). Low-quality sequences (PHRED < 20) and residual adapter sequences (stringency 1) were removed using Trim Galore (24), retaining only reads with a minimum length of 75 base pairs after trimming. The processed sequences were subsequently re-evaluated with FastQC v 0.12.1 to verify compliance with the required quality standards.

Once the quality of the cleaned sequences was confirmed, STAR v.2.7.10b (25) was used to align the clean reads (R1 and R2) from each sample against the reference genome of *Anisakis simplex* PRJEB496 (WormBase). This alignment allowed for the quantification of reads mapping to each gene in the reference genome, reflecting the relative abundance of each transcript in the analyzed samples. After obtaining the read counts per gene, differential gene expression analysis was conducted using the R package DESeq2 (26), considering as differentially expressed those genes with an adjusted *p-*value < 0.05, corrected using the Benjamini–Hochberg method. The comparisons were performed using pairwise comparisons between each experimental day and the control (day 0) under both 15 and 28 °C conditions.

From the set of differentially expressed genes (DEGs), volcano plots (R package EnhancedVolcano) were generated to visualize the relationship between the magnitude of gene expression changes and their statistical significance. Additionally, heatmaps were constructed to represent gene expression levels across different treatment conditions. To facilitate subsequent analyses, a preliminary gene filtering step was performed. Only genes showing and absolute log_2_fold change greater than |log_2_fold change| >2 were retained, in order to focus on the most strongly regulated and potentially biologically meaningful transcripts.

Enrichment analyses were performed on the filtered genes using G: Profiler (27), applying a Benjamini–Hochberg *padj* < 0.05. Enriched biological processes, molecular functions, and cellular components were visualized with bubble plots (all terms) and chord diagrams (top 10 GO terms and 40 most relevant genes) using SRplot (28). Additionally, the 25 most up- and downregulated genes were identified for each comparison. To gain further insights, orthologs in *Caenorhabditis elegans* PRJNA13758 were retrieved to explore potential metabolic pathways.

## 3 Results

### 3.1 Survival experiment

Throughout the experimental period, temperature remained stable according to set conditions, while pH showed only minor fluctuations, maintaining values near to pH 8 (± 0.1) At 15 °C, *Anisakis* L3 larvae remained viable until day 30, after which mortality began to occur progressively. By day 60, only a few larvae remained alive, most of which appeared dead. The experiment was therefore stopped at day 60. Accordingly, transcriptomic profiling was performed at days: 3 (D3), 7 (D7), 15 (D15), 30 (D30), and 60 (D60), each compared to the baseline control day cero. Conversely, at 28 °C all *Anisakis* larvae barely remained viable for a week (Supplementary Figure S1).

### 3.2 Overall differential expression genes

Differentially expressed genes (DEGs) were identified by pairwise comparisons between each sampling time point and the baseline control (D0) within each temperature treatment (15 and 28 °C). A substantial number of differentially expressed genes (DEGs) across each day (D3, D7, D15, D30, and D60) relative to the control (D0) at both 15 and 28 °C were detected (Supplementary Figures S2, S3, Table 1).

**Table 1:** Total and filtered numbers of differentially expressed genes (DEGs) identified in the experiments conducted at 15 and 28 °C.

**Temperature**	**Comparison**	**Total genes**	**Total filtered genes (%)**
15 °C	D3 vs. C	5,128	241 (5)
	D7 vs. C	5,872	241 (4)
	D15 vs. C	6,295	325 (5)
	D30 vs. C	6,360	399 (6)
	D60 vs. C	7,814	616 (8)
28 °C	D3 vs. C	6,335	460 (7)
	D7 vs. C	5,958	511 (8)

Comparisons were performed between each sampling time point and the baseline control (C = day 0). Sampling codes indicate days after the start of the experiment as follows: D3, day 3; D7, day 7; D15, day 15; D30, day 30; D60, day 60. Filtered genes correspond to annotated DEGs with an absolute fold change threshold of |log_2_FoldChange| ≥2. Percentages indicate the proportion of filtered DEGs relative to the total number of DEGs identified in each comparison.

**Figure 1:**
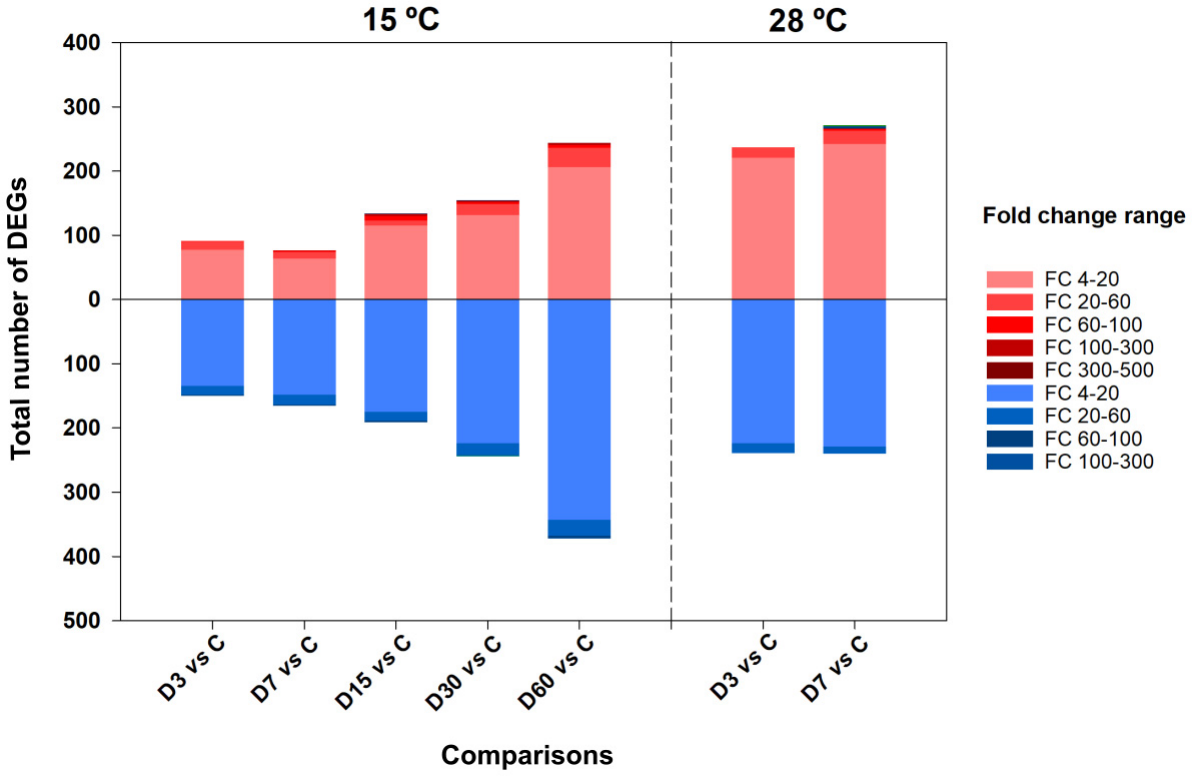
Number DEG (|log_2_FC|≥2) obtained on each comparison according to each intensity range expressed in fold change. Red bars represent the number of upregulated DEGs while bars in blue downregulated.

Following the filtering process, which involved retaining genes with a fold change greater than 4 compared to the control (i.e., |log_2_fold change| >2), between 241 and 616 DEGs were identified in the experiment conducted at 15 °C, depending of each comparison. At 28 °C, 460 DEGs were retained in the D3 vs. D0 comparison and 510 DEGs in the D7 vs. D0 comparison (Table 1, Supplementary Table S1). At 15 °C, the number of differentially expressed genes progressively increased over time throughout the survival experiment, reaching its peak at D60 (616 DEGs), with most genes displaying fold changes between 4- to 60-fold either up- or downregulated relative to the control (D0). In contrast, at 28 °C, the number of DEGs detected at D3 and D7 was substantially higher than at the corresponding time points at 15 °C, with the majority of expression changes still fell within the 4- to 60-fold change range compared to the control (Figure 1).

### 3.3 Comparative of Gene Ontology (GO) enrichment analysis

Functional enrichment analyses conducted on DEGs from the experiments at 15 and 28 °C revealed notable differences in functional enrichment between the two temperature conditions. Additionally, within each experiment, variations were observed on each sampling day.

#### 3.3.1 Gene Ontology (GO) terms at 15 °C

**Figure 2:**
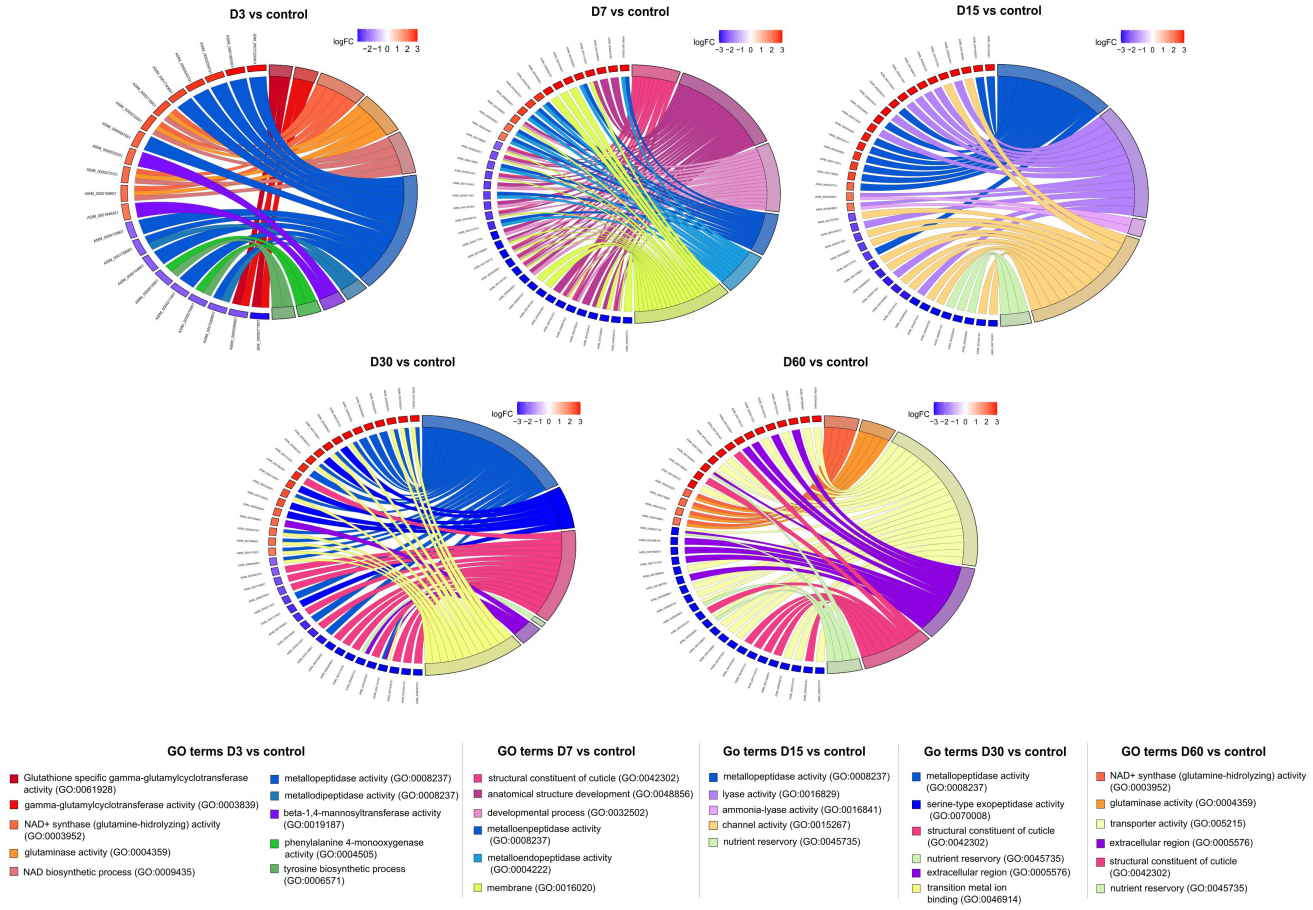
Chord diagrams illustrating the relationships between enriched Gene Ontology (GO) terms and the associated genes at each experimental time point (15 °C), in comparison with the controls (D0). Upregulated genes are shown in red and downregulated genes in blue.

Gene Ontology (GO) analysis revealed enrichment in terms related to structural development, peptidase activity, ion channel function, and glutamate metabolism across multiple time points vs. the control group (D0; Figure 2). GO terms associated with developmental processes (GO:0032502), anatomical structure development (GO:0048856), and structural constituents of cuticles (GO:0042302) were mainly enriched in D7, D30, and D60, primarily linked to downregulated genes (cuticlins, collagens). Peptidase-related GO terms, including metalloendopeptidase (GO:0008233, GO:0004222) and carboxypeptidase (GO:0004185, GO:0004180, GO:0008236) activity, were enriched from D3 to D30 and showed both up- and downregulation depending on the time point. Ion channel-related terms, such as channel activity and passive transmembrane transport, were enriched between D15 and D60, mainly associated with downregulated genes. However, upregulation of some ion channel-related functions was observed at D60 (e.g., glutamate receptors). Additional enriched terms included glycogen degradation (D15), nutrient reservoir activity [(GO:0045735) D15–D60, mostly downregulated], and glutamate metabolism (notably in D3), including glutaminase and NAD^+^ synthase activity. Enrichment in tyrosine biosynthesis (GO:0006571, GO:0004505, GO:0019293, GO:0009095) and glutathione degradation [(GO:0061928; gamma-glutamylcyclotransferase activity] was also observed in D3, generally associated with downregulated gene expression (Figure 2).

#### 3.3.2 Gene Ontology (GO) terms at 28 °C

**Figure 3:**
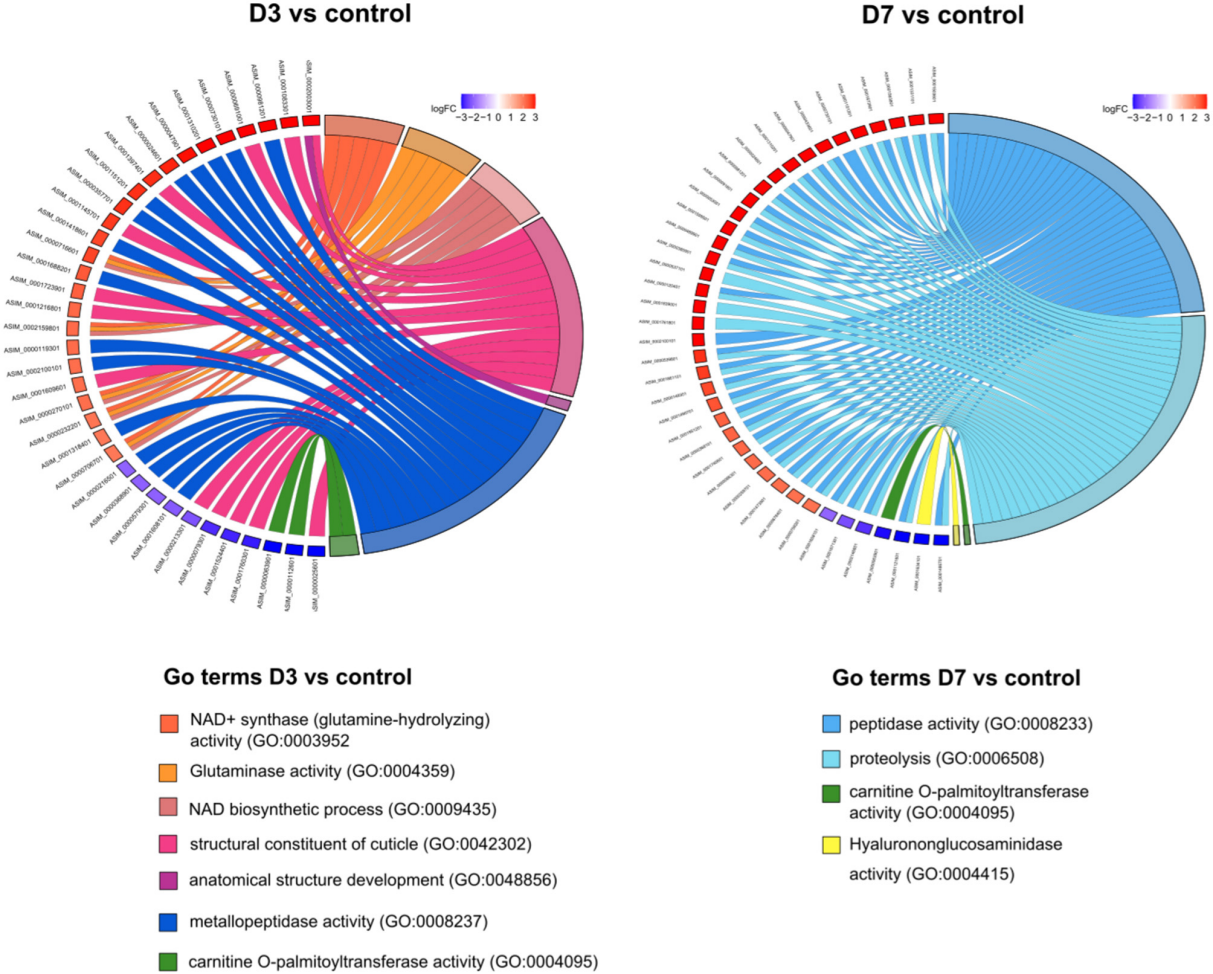
Chord diagrams illustrating the relationships between enriched Gene Ontology (GO) terms and the associated genes at each experimental time point (28 °C), in comparison with the controls (D0). Upregulated genes are shown in red and downregulated genes in blue.

As observed at 15 °C, Gene Ontology (GO) terms related to nicotinamide (NAD^+^) biosynthesis and glutaminase activity were among the most enriched at D3. These included glutaminase activity (GO:0004359), NAD^+^ synthase (glutamine-hydrolyzing; GO:0003952), and pyridine-containing compound biosynthetic process (GO:0072525), among others (Figure 3). A moderate enrichment of NAD^+^ synthase activity (GO:0003952) was also detected at D7. In contrast to the 15 °C condition, GO terms associated with cuticle structural components (GO:0042302), developmental processes (GO:0032502), and anatomical structure development (GO:0048856) were significantly enriched at D3, but not at D7. Additionally, GO terms related to proteolysis (GO:0006508) and peptidase activity (GO:0008233) were enriched in both D3 and D7, with greater representation at D7 (Figure 3).

### 3.4 Structural cuticle-related genes

**Figure 4:**
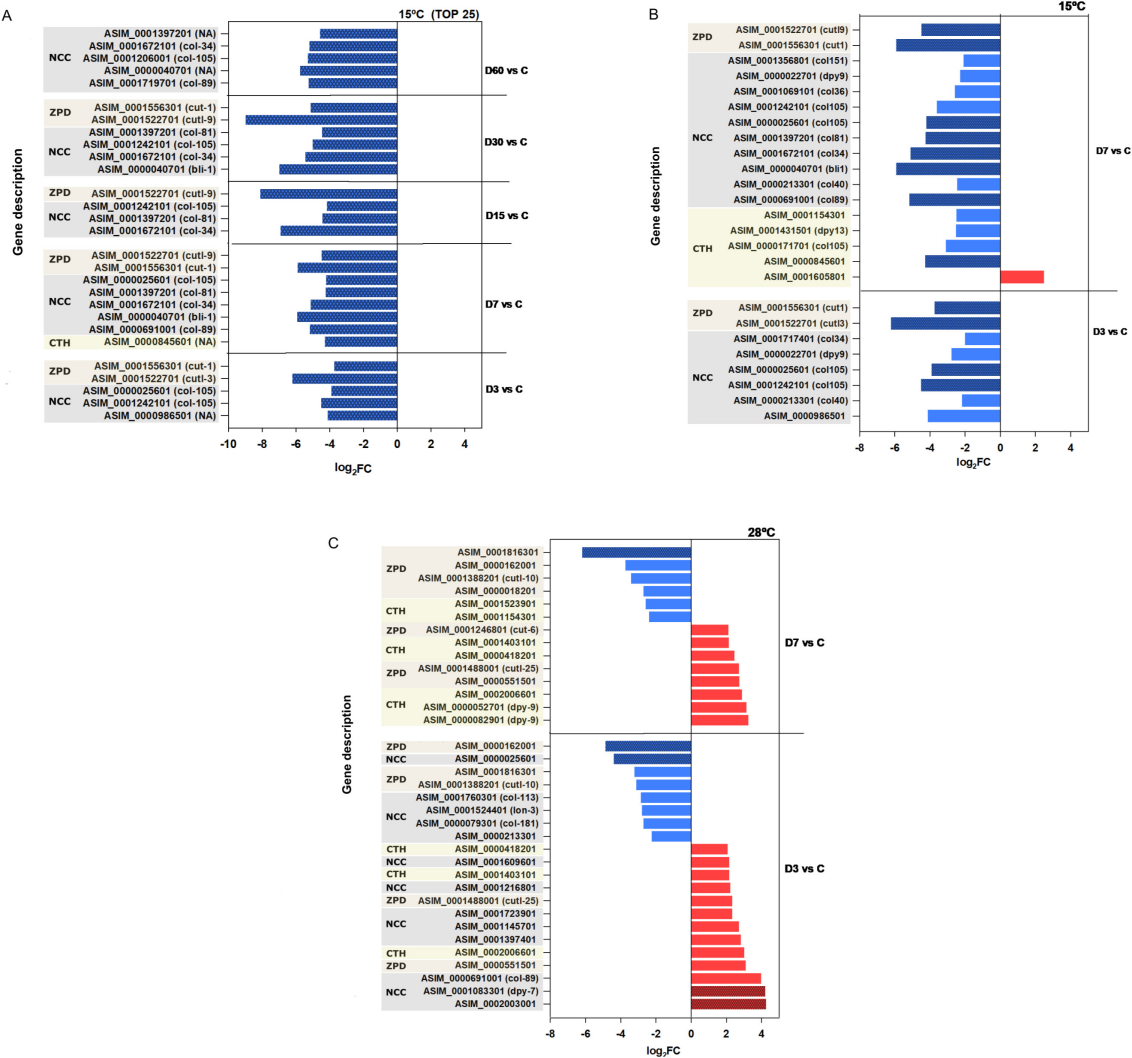
Expression levels (log_2_FC) of structural cuticle related DEGs at 15 and 28 °C. **(A)** Genes identified at 15 °C that were among the top 25 most downregulated genes in each comparison. **(B, C)** Expression profiles of at day 3 (D3) and day 7 (D7) under 15 °C **(B)** and 28 °C **(C)** conditions, highlighting temporal changes in gene regulation at both temperatures. Red bars indicate upregulated genes, and blue bars indicate downregulated genes. Dark-colored bars with patterns highlight genes ranked among the top 25 most upregulated or downregulated in each comparison, based on the total set of differentially expressed genes. NCC, nematode cuticle N-terminal; CTH, collagen triple helix; ZPD, zona pellucida domain.

Differentially expressed genes (DEGs) related to cuticle structure were consistently represented in larvae incubated at both 15 and 28 °C across all time points (Figure 4). These genes included those annotated with domains such as collagen triple helix, nematode cuticle N-terminal collagen, and pellucid zone. Their *Caenorhabditis elegans* orthologs correspond to cuticle and collagen related genes (e.g., *dpy-9, dpy-13, col-34, col-81, col-105*), cuticlins (*cut-1*), and cuticlin-like genes (*cutl-3, cutl-9*; Figure 4A, Supplementary Table S1). Notable differences in gene expression patterns were detected between the two temperature conditions. At 15 °C, the majority of cuticle-related transcripts were downregulated from D3 to D60 relative to control (D0), with several consistently ranking among the TOP25 most downregulated genes. These transcripts exhibited substantial reductions in expression, with log2 fold change (log_2_FC) values ranging from −3 to −8 (Figure 4B; Supplementary Table S2). In contrast, at 28 °C, both upregulated and downregulated transcripts were detected as early as D3 and D7. Some genes reached log_2_FC values of +4 and were included among the top 25 most upregulated transcripts (Figure 4C; Supplementary Table S1).

### 3.5 Peptidase activity related genes

The expression of genes encoding peptidases was significant at both temperatures across all experimental comparisons. However, a higher number of peptidase DEGs was observed at 28 °C, with most of them being upregulated relative to the control condition (Supplementary Figure S4).

**Figure 5:**
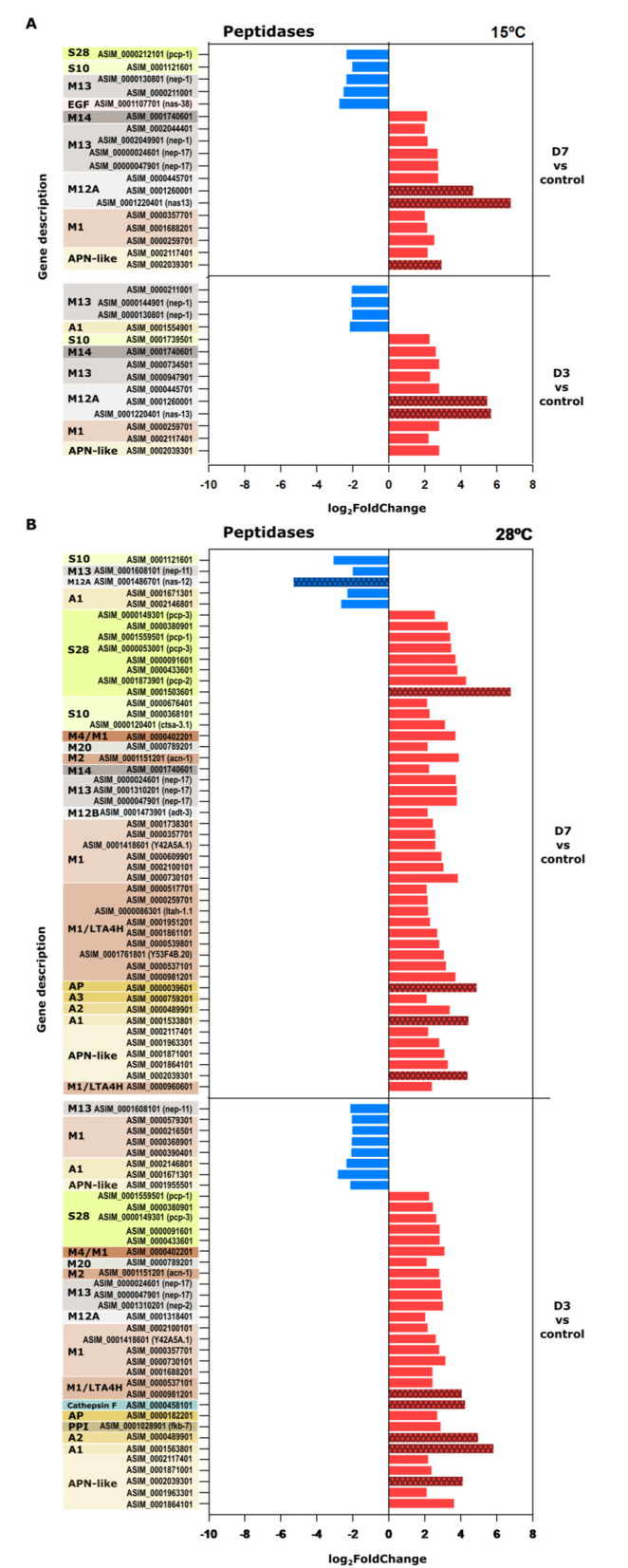
Expression levels (log_2_FC) of peptidase related genes at 15 and 28 °C. **(A)** Genes identified at 15 °C for D3 and D7 relative to the control. **(B)** Gene expression at 28 °C for D3 and D7 relative to the control. Red bars indicate upregulated genes, while blue bars denote downregulated genes. Red bars indicate upregulated genes, and blue bars indicate downregulated genes. Dark-colored bars with patterns highlight genes ranked among the top 25 most upregulated or downregulated in each comparison, based on the total set of differentially expressed genes. Serin proteases: S10, S28; metallopeptidases: M1, M2, M4 M12, M12A, M13, M14, M20 M1/LTA4H (M1- leukotriene A4 hidrolase); aspastic proteases (AP): A1, A2, A3, APN-like (aspartic protease N like).

At 15 °C, peptidase gene expression displayed a time-dependent increase, with the number of DEGs rising progressively from 15 at D3 to 30 at D60 with an average of 3 log_2_FC. Among the most prominent peptidase gene families at this temperature were those encoding aspartic peptidades (A1), aminopeptidases (M1), carboxypeptidades (M14, S10, S28; particularly *pcp-1*) and metallopeptidases of families M12 (*nas-13*) and M13 (*nep-1, nep-17*). These genes were predominantly upregulated compared to the control (Supplementary Table S2) and several were included among the top 25 most upregulated DEGs (as *nas-13* and *nas-38 C. elegans* orthologs) with the exception of D60 (Figure 5A, Supplementary Figure S4).

At 28 °C the response was both more pronounced and more immediate. A broad spectrum of peptidase-encoding genes was differentially expressed as early as D3 with 37 peptidase DEGs and at D7 with 49 DEGs. They include encode aspartic peptidades (A1), aminopeptidases (M1), leukotriene A4 hydrolases, carboxypeptidades [M14, S10, S28 (*pcp-1*), metallopeptidases M12: *nas-13*; M13: *nep-1, nep-17*] and Cathepsin F (papain C1A; Figure 5B). Many of these transcripts were among the top 25 most upregulated DEGs in both the D3 vs. control and D7 vs. control comparisons with log_2_FC values ranging from 2 to 6 (Figure 5B, Supplementary Table S3). For instance, highly expressed transcripts included Peptidase A1 (ASIM-0001563801), aminopeptidase (ASIM-0002039301), cathepsin F (ASIM-0000458101), and peptidase S8 (ASIM-1503601), indicating a robust early response to elevated temperature (Figure 5B).

### 3.6 Metabolism related genes

Genes involved in gluconeogenesis (phosphoenolpyruvate carboxykinase PEPCK, *pck*-3 (*C. elegans* ortholog), and, galactose metabolism (Alpha galactosidase *gana-1*) were consistently upregulated across all comparisons at 15 °C, some of them among the top 25 most upregulated transcripts (Table 2). In contrast, under the 28 °C condition, these genes were not differentially expressed. Conversely, genes encoding malate-L-lactate dehydrogenase [tricarboxylic acid (TCA) cycle], sugar transporters of the SWEET family, and aldolases (TIM barrel aldolases) were consistently found among the top 25 most downregulated genes across all comparisons relative to the control, both at 15 and 28 °C (Table 2). Additionally, marked downregulation was observed in genes encoding trehalases, such as *tre-3*, particularly in the D15 vs. control (ASIM_0000603401) and D60 vs. control (ASIM_0001878601) comparisons conducted at 15 °C, although the intensity of downregulation remained within a range of log_2_FC < 3.

**Table 2:** Differentially expressed genes associated with carbohydrate metabolism included among the top 25 upregulated and downregulated genes in all comparisons performed at 15 and 28 °C.

**T°(°C)**	**Comparison**	**Transcript description**	**ID**	**Log_2_FC**	**Padj**	**Uniprot ID**	**Orth. *C. elegans***
15	D3 vs. control	Glycoside hydrolase family 27	ASIM_0000510801	3.4	0.0013	A0A0M3JBY0	*gana-1*
		Phosphoenolpyruvate carboxykinase GTP-utilizing	ASIM_0001745401	4.02	0.0104	A0A0M3K912	*pck-3*
		Aldolase-type TIM barrel	ASIM_0001634101	−4.98	0.0004	A0A0M3K5V0	*NA*
	D7 vs. control	Glycoside hydrolase family 27	ASIM_0000510801	3.18	0.007	A0A0M3JBY0	*gana-1*
		Phosphoenolpyruvate carboxykinase GTP-utilizing	ASIM_0001688701	3.96	0.0013	A0A0M3K7E6	*pck-3*
		Aldolase-type TIM barrel	ASIM_0001634101	−4.92	0.0005	A0A0M3K5V0	*NA*
		SWEET sugar transporter	ASIM_0001534101	−5.26	0.002	A0A0M3K311	*swt-3*
	D15 vs. control	Glycoside hydrolase family 27	ASIM_0000510801	3.89	0.0002	A0A0M3JBY0	*gana-1*
		Phosphoenolpyruvate carboxykinase GTP-utilizing	ASIM_0001688701	4.67	0.0001	A0A0M3K7E6	*pck-3*
		Phosphoenolpyruvate carboxykinase GTP-utilizing	ASIM_0000411001	3.98	0.0124	A0A0M3J949	*NA*
		Phosphoenolpyruvate carboxykinase GTP-utilizing	ASIM_0001745401	3.96	0.0253	A0A0M3K912	*pck-3*
		SWEET sugar transporter	ASIM_0001534101	−4.52	0	A0A0M3K311	*swt-3*
	D30 vs. control	Phosphoenolpyruvate carboxykinase GTP-utilizing	ASIM_0001688701	4.45	0.0001	A0A0M3K7E6	*pck-3*
		Phosphoenolpyruvate carboxykinase GTP-utilizing	ASIM_0001745401	4.39	0.0036	A0A0M3K912	*pck-3*
		SWEET sugar transporter	ASIM_0001534101	−3.77	0	A0A0M3K311	*swt-3*
28	D3 vs. control	Malate/L-lactate dehydrogenase-like	ASIM_0000320501	−3.95	8.589E-50	A0A0M3J6L6	F36A2.3
		Malate/L-lactate dehydrogenase-like	ASIM_0002125001	−4.18	9.745E-29	A0A0M3KJS4	NA
		Malate/L-lactate dehydrogenase-like	ASIM_0002081501	−4.20	5.768E-23	A0A0M3KIJ6	F36A2.3
		SWEET sugar transporter	ASIM_0001534101	−4.40	1.29337E-55	A0A0M3K311	*swt-3*
	D7 vs. control	Aldolase-type TIM barrel	ASIM_0001634101	−4.10	3.6E-05	A0A0M3K5V0	NA
		Malate/L-lactate dehydrogenase-like	ASIM_0002081501	−4.10	7.97E-21	A0A0M3KIJ6	F36A2.3

For each gene, the description, expression magnitude (log_2_FoldChange), adjusted p-value (padj), corresponding UNIPROT protein identifier, and the orthologous gene in *Caenorhabditis elegans* are provided.

**Figure 6:**
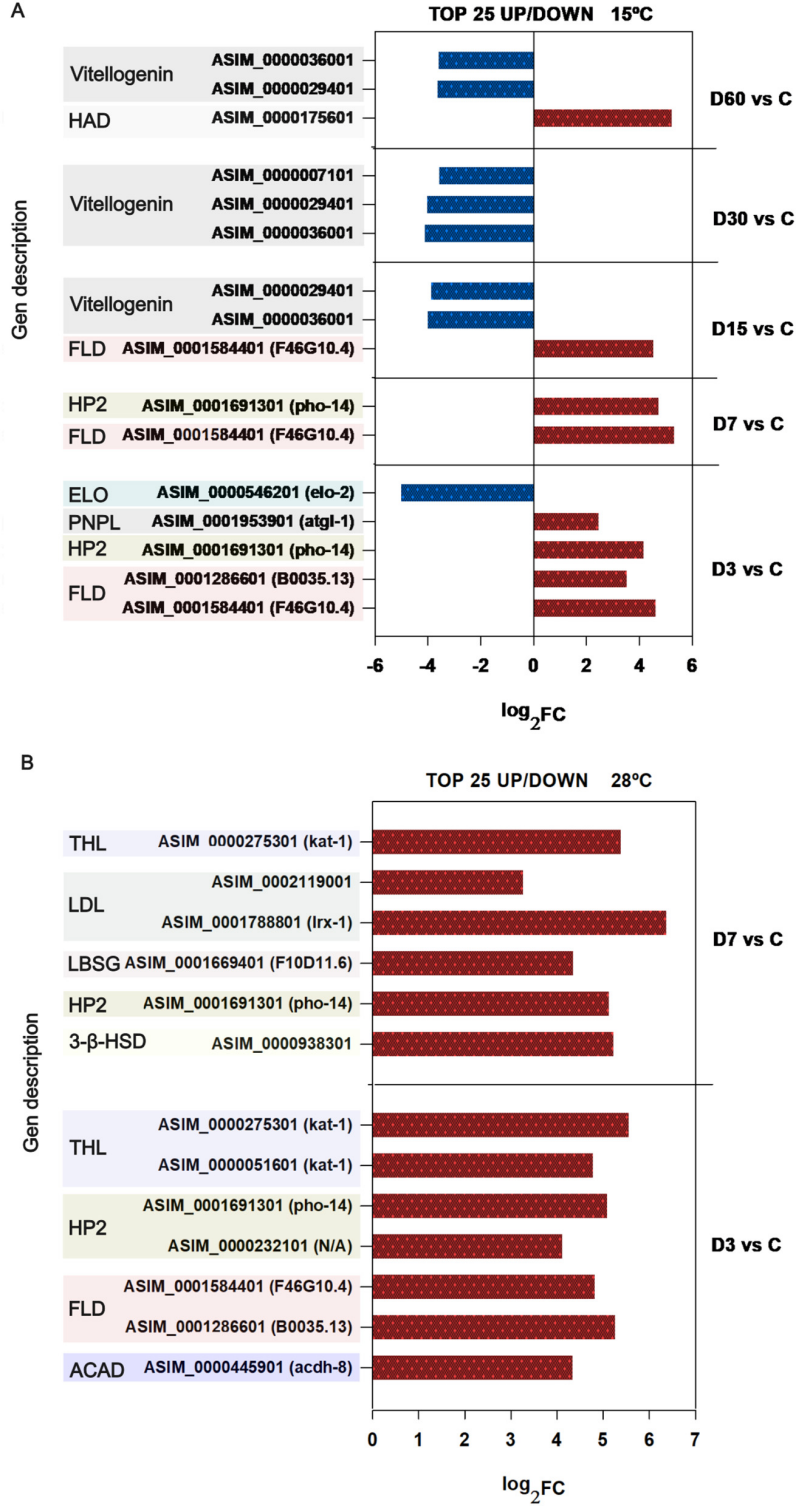
Expression levels (log_2_FC) of lipid metabolism-related genes at 15 and 28 °C. Genes associated with lipid metabolism that rank among the 25 most differentially expressed (upregulated or downregulated) are shown at 15 °C **(A)** and 28 °C **(B)**. Red bars indicate upregulated genes, and blue bars indicate downregulated genes. HAD: hidrolase superfamily HAD; FLD: fungal lipase like domain; HP2: histidine phosphatase superfamily 2; ELO: elongase; PNP: patatin like phosphatase; THL: thiolase like domain; LDL: low density lipoprotein receptor class A; LBSG, lipid binding serum glycoprotein C-terminal; ACAD: Acyl-CoA dehidrogenase.

Differentially expressed genes (DEGs) related to lipid synthesis, storage, and lipolysis were notably prevalent at 15 °C, with several appearing among the top 25 upregulated or downregulated genes (Figure 6A). Genes involved in lipid synthesis such as, those encoding elongases (*elo-2, elo-4*), were markedly downregulated across all comparisons performed at 15 °C. In the D3 vs. control comparison, *elo-2* was listed among the top 25 most downregulated transcripts (log_2_FC = −5). At 28 °C, genes involved in lipid biosynthesis were not represented among the 25 most up or downregulated genes (Figure 6B).

The most representative transcripts at both temperatures were those associated with lipid degradation: triacylglycerol breakdown and acid β-oxidation, the majority of which ranked among the top 25 most upregulated genes in both experiments. At 15 °C, overexpressed genes included lipases (*lip-3*) and phospholipases (*atgl-1*), consistently upregulated under all conditions, with expression intensities ranging between log_2_FC 3 and 5. At 28 °C, in addition to these, transcripts encoding thiolases (*kat-1*) and acyl-CoA dehydrogenase (*acdh-8*) were also prominently upregulated, appearing among the top 25 most overexpressed genes in both comparisons performed (D3 vs. control and D7 vs. control; Figure 6).

Furthermore, transcripts associated with vitellogenin precursors, known to play a crucial role in lipid storage, were among the top 25 most downregulated genes in the later stages of the experiment conducted at 15 °C (D15–D60 vs. control). Conversely, at 28 °C, several genes implicated in cholesterol homeostasis were found to be upregulated (Figure 6).

### 3.7 Neuronal related genes

**Figure 7:**
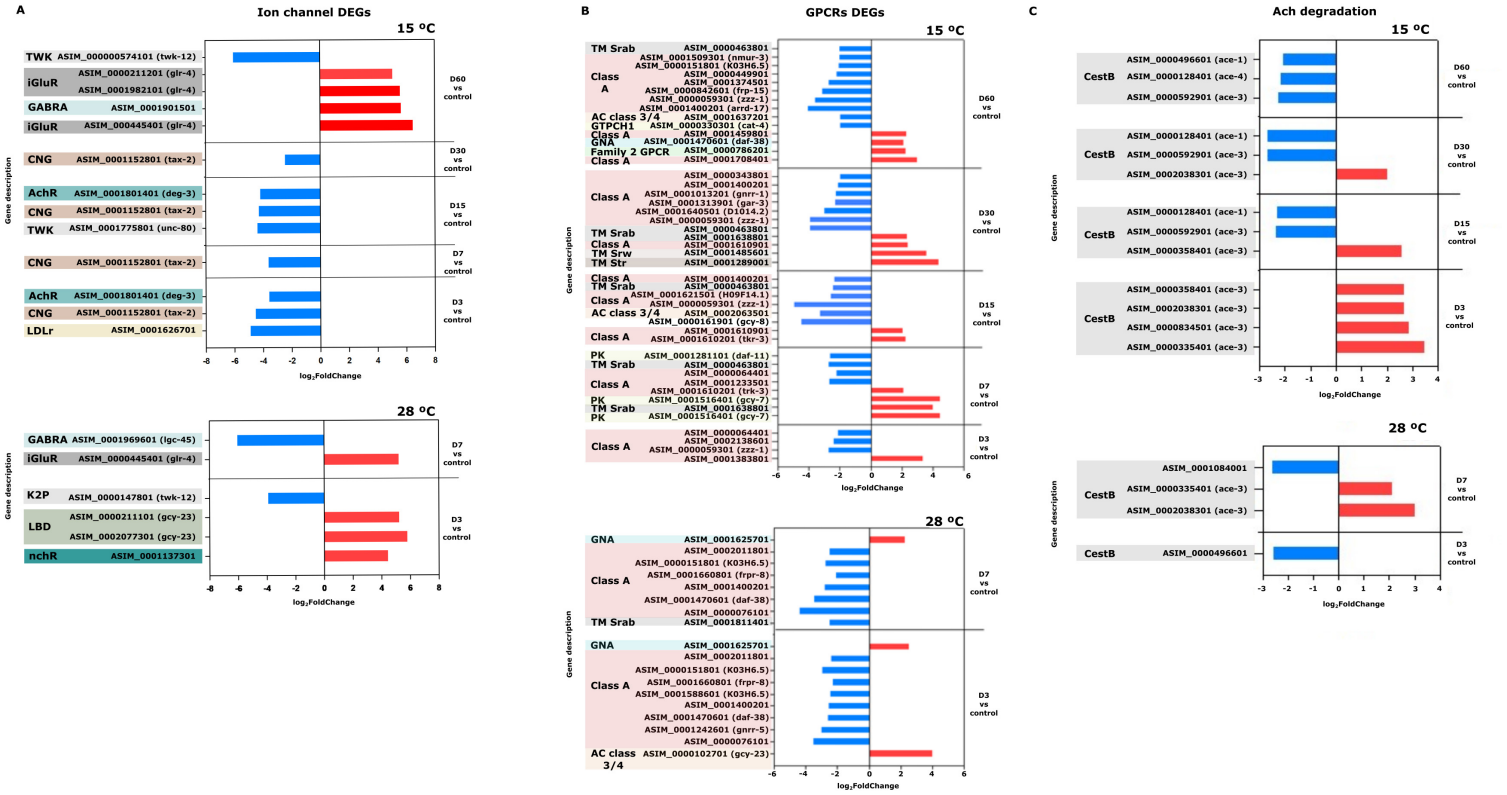
Expression levels (log_2_FC) of neuronal related genes at 15 and 28 °C. Red bars indicate upregulated genes, and blue bars indicate downregulated genes. **(A)** Genes involved in ion channel function. TWK/K2P: two pore domain potassium channel; iGlur: ionotropic glutamate receptor C-terminal; GABRA: Gamma-aminobutyric acid A Glycine receptor Alpha; CNG: cyclic nucleotide binding domain; nchR: neurotransmiter gated-ion channel transmembrane domain; LBD: receptor ligand binding domain. **(B)** G protein-coupled receptors (GPCRs) TM Srab: TM GPCR serpentine receptor class ab; TM Srw: TM GPCR serpentine receptor class w; TM Str: TM GPCR serpentine receptor class r; Class A: GPCR rhodopsin-like receptor; AC class ¾: adenylil cyclase class ¾ guanilyl cyclase; GTPCH1: GTP cyclohidrolase I; GNA: Guanine nucleotide binding protein (G protein) alpha subunit; PK: protein kinase. **(C)** Acetylcholine degradation related DEGs. CestB: carboxylesterase type B.

Genes involved in ion channel function were identified, most of which were found to be downregulated across all comparisons and in both experiments. Notably, this included genes associated with the maintenance of potassium homeostasis (*kcc-3, unc-93*, members of the TWK family, *kqt-1*), chloride homeostasis (*best-14, clh-3, clh-2*), and sodium balance (*del-6, del-9, deg-3*). An exception was *unc-58*, a TWK family gene, which was upregulated at 15 °C in all comparisons. Additionally, low expression levels were observed in genes related to cyclic nucleotide-gated (CNG) ion channels, such as *spe-41, tax-2*, and *tax-4*, as well as in those activated by neurotransmitter binding (Figure 7A). Neurotransmitter-gated ion channels were also differentially expressed in both the 15 and 28 °C experiments. Acetylcholine-gated ion channels (*deg-3, des-2*) were downregulated in all comparisons (D3–D60) at 15 °C. Similarly, gamma-aminobutyric acid (GABA)-gated ion channel transcripts (*lgc-44, lgc-45, lgc-46, mod-1*) were largely downregulated (with some appearing among the top 25 most downregulated genes) in both temperature conditions. The exception was the gene ASIM_0001901501, which encodes a gamma-aminobutyric acid (GABA)-regulated ion channel, was observed. This gene was among the 25 most highly overexpressed genes in the comparisons of D30 vs. control and D60 vs. control at 15 °C. Similarly, significant overexpression of genes encoding ionotropic glutamate receptors (*glr-4*) was detected; these were also ranked among the 25 most overexpressed genes in the D60 vs. control comparison at 15 °C, as well as in both comparisons performed at 28 °C.

G protein-coupled receptors (GPCRs; *tkr-3, zzz-1, gar-3, gnrr-1*), along with genes encoding G proteins and guanylyl cyclases (*daf-11*), also exhibited differential expression across all time points. At 15 °C, these genes displayed both up- and downregulation depending on the time point, whereas at 28 °C, they remained consistently downregulated in both comparisons (Figure 7B).

A differential expression pattern was observed in genes involved in acetylcholine degradation (orthologs of *C. elegans*: *ace-1, ace-2, ace-3*, and *ace-4*). Most of these genes were upregulated in the D3 vs. control comparison, but their expression levels decreased markedly from D15 to D60 compared to the control at 15 °C. Notably, *ace-3* was among the 25 most highly upregulated genes on D3. In contrast, in the experiment conducted at 28 °C, ace-3 also exhibited upregulation on day D7 (Figure 7C).

## 4 Discussion

The experimental evaluation of the contrasting potential impact of current and heated extreme temperatures (comparable to those occurring during marine heatwaves) on the survival and transcriptomic signature of free-living third-stage larvae of *A. simplex* provided significant insights.

Firstly, the epidemiological perspective over the past 25 years sheds light on how the Mediterranean sibling species (*A. pegreffii*) has steadily expanded into the colder Atlantic waters of the Iberian Peninsula (18). This poleward shift likely reflects northward host migration of cetaceans, fishes and invertebrates in response to rising sea surface temperature (29–31), but the borealization of *Anisakis* life cycle could also be further modulated by increasingly frequent marine heatwaves events affecting larval survival and fitness. After being released with the feces of the definitive host, *Anisakis* eggs develop in the marine environment and hatch into free-swimming larval stages that occur predominantly in the water column before entering the marine food web. Additionally, along the Atlantic Spanish coast the free-living L3 may be also found floating in the surface when they are discarded by fishing fleet with fish offals, thus entering the subsurface water where L3 are commonly fed on by zooplankton (32). Therefore, subsurface free-living L3 may be severely exposed to marine heatwaves (11), and their level of impact markedly depends on the heat tolerance range of the parasitic larvae. In fact, *A. simplex* was not frequently cited in warm waters such as those of the Mediterranean Sea (15), as it seems likely that it cannot survive the frequency and duration of marine heatwaves currently recorded at the Mediterranean. Our experimental findings indicate that *A. simplex* may exhibit a marked transcriptomic response under real marine heatwaves scenarios, which is revealed from both survival patterns and underlying molecular adaptations to thermal variation. Parasitic larvae maintained at 15 °C survived for up to 60 days, in contrast to those exposed at 28 °C, where survival did not exceed 1 week. Transcriptomic profiles across multiple time points at 15 °C showed a progressive increase in differentially expressed genes (DEGs), suggesting the activation of adaptive and stress tolerance pathways over time. Conversely, exposure to 28 °C resulted in a rapid and pronounced increase in DEGs in just 1 week, indicative of acute thermal stress and reduced physiological resilience. These results are consistent with previous comparisons of thermal tolerance between *A. simplex* and *A. pegreffii* during early larval stages (33). Therefore, the data suggest that cooler seawater not only promotes larval viability but also drives a transcriptional modulation that may increase the likehood of transmission to host, ultimately reinforcing the parasite's persistence and epidemiological potential in marine ecosystems.

The physiological basis of the above-mentioned responses seems clearly a challenge gap to *Anisakis* life-cycle under a One Health approach (34). Therefore, we further examined key functional categories of differentially expressed genes (DEGs). Each category provided insights into how *A. simplex* modulates its physiology and development in response to adverse challenge environmental conditions.

### 4.1 Structural cuticle-related genes

Given the critical role of the cuticle in nematodes in both larval protection and the molting process (35, 36), the transcriptomic profile of *A. simplex* in seawater revealed distinct modulation of cuticle related genes. At 15 °C, most genes involved in cuticle formation, mainly collagens and cuticlins, showed sustained downregulation from all the study period (D3–D60), suggesting a developmental arrest. This is likely due to the absence of definitive host-like conditions (e.g., 37 °C, 5% CO_2_) that are known to trigger the transition from the L3 to the adult stage. This lack of activation is consistent with previous transcriptomic studies showing that, in the absence of definitive host-like condition, *Anisakis* larvae fail to initiate molting-related processes (4, 20). In contrast, at 28 °C, cuticle-related genes displayed a heterogeneous expression pattern with both upregulation and downregulation at early time points (D3–D7). Notably, two of the most highly upregulated genes at D3 (*dpy-7, dpy-9*) are directly involved in cuticle structure, suggesting that larvae may initiate partial activation of the molting process under warmer conditions. Similar temperature-driven partial activation of developmental and cuticle-associated genes has been reported in transcriptomic studies of *Anisakis simplex* and closely related species (4, 20). However, the overall pattern implies that these environmental conditions are still insufficient to support complete cuticle remodeling and full developmental progression.

### 4.2 Peptidase related genes

Peptidases are enzimes that catalyze the hydrolysis of peptide bonds in proteins. In parasitic nematodes, they play essential roles in a wide range of processes such as tissue invasion, development and molting, nutrient acquisition, immune evasion and adaptation to environmental stress (37).

At 15 °C, the gradual upregulation of peptidase related genes may suggest an adaptive response of *A. simplex* L3 larvae in order to maintain protein homeostasis by removing damaged or misfolded proteins to prolong survival under moderate thermal conditions. The upregulation of neprilysin genes (e.g., *nep-17*), particularly from day 7 to 60 in the experiment conducted at 15 °C and at both conditions performed at 28 °C, likely leads to enhanced peptide degradation, potentially impacting physiological processes dependent on these substrates. Altered peptide availability may influence cellular functions such as neuronal signaling, stress response, and other critical processes. Additionally, the prolonged upregulation of neprilysins could be linked to their neuroprotective role, enabling survival under sustained stress conditions (38).

In contrast, at 28 °C this parasite showed an intense activation and upregulation of genes associated with peptidase activity, which were among the most significantly enriched GO terms. These genes included genes encoding peptidase S8 family members and lysosomal proteases, such as cathepsins. Under stress conditions such as elevated temperature, lysosomal enzymes are known to promote necrotic cell death pathways, while members of the peptidase S8 family have been implicated in immune evasion responses (39). The marked decline in survival observed by day 7 at 28 °C support this assumption, suggesting a threshold beyond which the parasite's compensatory mechanisms collapse.

### 4.3 Metabolism related genes

Metabolic flexibility is a key adaptive feature in parasitic nematodes, allowing them to survive in unfavorable environmental conditions. Upon leaving the final host, *Anisakis* spp. must reprogram its metabolism to conserve energy and maintain cellular homeostasis. Our transcriptomic data show that third-stage larvae exhibit significant modulation of genes related to carbohydrate and lipid metabolism, reflecting a coordinated response aimed at conserving internal energy reserves and utilizing alternative carbon sources.

At both 15 and 28 °C, we detected a consistent downregulation of genes involved in sugar uptake (SWEET family transporters), glycolysis (TIM-barrel aldolases) and the tricarboxylic acid (TCA) cycle (malate/L-lactate dehydrogenases), indicating a broad suppression of carbohydrate metabolism. In turn, compensatory pathways appear to be activated, most notably gluconeogenesis. The persistent upregulation of phosphoenolpyruvate carboxykinase (PEPCK, *pck-3, C. elegans* ortholog) at 15 °C supports this shift. PEPCK enables glucose synthesis from non-carbohydrate sources, a mechanism commonly observed in stress-adapted stages like the dauer larva of the free-living nematode *C. elegans* (40). Further evidence for metabolic adaption is provided by the sustained overexpression of alpha galactosidase genes (*gana-1, C. elegans* ortholog), suggesting mobilization of galactosides as additional energy sources during early free-living phase. Moreover, the downregulation of trehalase genes at days 15 and 60 may facilitate the accumulation of trehalose, known to function both as an energy reserve and a protective molecule under stress conditions, including starvation and temperature changes already noted in *A. simplex* (41). Altogether, these transcriptional changes point to a strategic shift away from classical glucose metabolism toward stress-resilient pathways for energy conservation and survival, particularly at 15 °C, while higher thermal stress at 28 °C appears to suppress carbohydrate metabolism more broadly, suggesting a limited adaptive window under warmer conditions.

Lipid metabolism also emerged as a central component of the adaptative response of *A. simplex* larvae. Lipids not only serve as high-energy storage molecules but also contribute to structural integrity and signaling pathways (42, 43). Triacylglycerides (TAGs), are hydrolysed by lipases such as *atgl-1*, releasing fatty acids that are subsequently oxidized in mitochondria and peroxisomes via β-oxidation. This process generates acetyl-CoA, which fuels the TCA cycle and supports ATP production (43, 44).

In this study, the marked upregulation of genes involved in TAG degradation (*atgl-1, lip-3 C. elegans* orthologs), along with genes related to β-oxidation (*acdh-7*, thiolases), suggest a strong activation of lipid catabolic pathways. Several of these genes consistently ranked among the top 25 most upregulated transcripts at both 15 and 28 °C, highlighting a strong activation of lipid catabolism aimed at mobilizing internal energy reserves. Notably, lipase-3 is a lysosomal enzyme also associated with autophagy pathways, which are commonly upregulated during nutrient deprivation or cellular stress in C. *elegans* (43). Its expression suggests that *A. simplex* may rely not only on classical lipolysis but also on autophagy-mediated lipid mobilization to meet energy demands. In contrast, genes involved in lipid biosynthesis (e.g., elongases) and storage (e.g., apolipoproteins) were significantly downregulated, particularly at 15 °C. Lipogenesis is an energetically expensive process, and its suppression under stress likely reflects a shift toward energy conservation, prioritizing catabolism over anabolism.

Interestingly, the downregulation of *lip-3* at Day 60 (15 °C), and of both *lip-3* and carnitine O-palmitoyltransferase at Days 3–7 (28 °C), may signal a transition to a critical physiological state, possibly indicating progressive energy depletion. Notably, from day 15 onward, transcripts related with nutrient reserves, particularly vitellogenin- N- terminal genes, consistently ranked among the top 25 most downregulated genes. Vitellogenin's central role in lipid transport and energy storage, its suppression strongly reinforces the idea of a sustained loss of energy reserves during this period.

Supporting this interpretation, the stress-response genes such as *pgph-3* (a *C. elegans* phosphoglycolate phosphatase ortholog), was among the top 25 most upregulated transcripts at day 60 at 15 °C. This gene is known to promote stress tolerance and longevity (45), suggesting that larvae under extreme metabolic pressure may activate protective pathways to prolong survival.

Overall, the transcriptional profile of *A. simplex* larvae incubated at 15 and 28 °C, reveals a prioritization of lipid degradation over synthesis in order to enable energy mobilization to survive.

### 4.4 Neuronal related genes

Neuronal and signaling gene expression in *A. simplex* L3 exposed to 15 and 28 °C seawater reveals different temporal stress responses, highlighting both adaptive strategies and eventual failure under prolonged unfavorable conditions.

At 15 °C, early transcriptional changes suggest a neuroprotective strategy aimed at conserving energy and maintaining environmental sensitivity. Key ion channel genes, including potassium channels (TWK family), chloride channels (bestrophin), cyclic nucleotide-gated channels (*tax-2*), and Transient Receptor Potential (TRP) channels (*spe-41*) related genes, were significantly downregulated. This general suppression, also observed in *C. elegans* under environmental stress, may impair sensory perception and osmoregulation (46–48). Strikingly, this transcriptional profile shares a certain parallelism with the activation of dauer entry programme in *C. elegans* (49), in which downregulation of GPCRs and *daf-11* (a guanylyl cyclase) leads to reduced cGMP signaling and developmental arrest (50). In *A. simplex*, the downregulation of ortholog genes of *daf-11*, GPCRs, and their downstream ion channels, together with upregulation of the sensory guanylyl cyclase *gcy-7*, suggests a shift from growth-promoting signals to a survival-oriented, sensory-alert state. This may represent a dauer-like regulatory state, where larvae reduce metabolic expenditure while remaining responsive to environmental changes. This is further supported by the persistent downregulation of transcripts whose *C. elegans* ortholog is the acetylcholine receptor subunit gene *deg-3*, which has been associated with excitotoxicity in *C. elegans*.

In this organism model, upregulation of *deg-3* produces calcium-mediated neuronal death, while its suppression limits excitatory drive. Thus, downregulation of ortholog genes of *deg-3* in *A. simplex* may act as a neuroprotective mechanism to minimize synaptic excitation and neuronal damage, since gain of function of degenerins promotes necrotic cell death events (39). Complementing this response, we also observed early upregulation of acetylcholinesterase AChEs genes (*ace-1, ace-2, ace-3, ace-4 C. elegans* orthologs). Acetylcholine is the main excitatory neurotransmitter, but its activity must be tightly regulated to prevent overstimulation of cholinergic receptors, achieved through the rapid degradation of acetylcholine by acetylcholinesterases (AChEs). The observed upregulation in early day times may be possibly to enhance degradation of acetylcholine and fine-tune cholinergic signaling during initial adaptation (51, 52). Together, these patterns suggest an active dampening of neuronal excitability to preserve integrity during early environmental exposure.

However, this adaptive state appears unsustainable over time. At later time points (days 30–60), parasitic larvae incubated at 15 °C exhibited upregulation of genes similar to *unc-58* (potassium channel) and *glr-4* (AMPA-type glutamate receptor), genes whose overexpression in *C. elegans* is linked to motor dysfunction and excitotoxic neuronal damage due to calcium influx (53, 54). Moreover, the downregulation of AChE genes at these stages could further exacerbate cholinergic imbalance, leading to acetylcholine accumulation and sustained depolarisation, both of which may impair neuronal function (51, 52). By contrast, at 28 °C, signs of excitotoxic dysregulation emerged earlier from day 3. The overexpression of *glr-4* and *unc-58* was already evident at days 3–7, and AChE genes were downregulated from the start. This suggests that thermal stress at 28 °C triggers immediate neuronal over activation, bypassing the earlier neuroprotective phase seen at 15 °C and accelerating the onset of dysfunction.

In summary, *A. simplex* L3 larvae show an initial neuroprotective strategy at 15 °C, characterized by suppression of excitatory signaling and partial resemblance to dauer-like regulation, but prolonged stress leads to neuronal dysregulation. At 28 °C, excitotoxicity emerges rapidly, suggesting reduced adaptive capacity. This reflects a delicate balance between stress adaptation and neural stability.

## 5 Conclusion

This study reveals that *A. simplex* third-stage larvae (L3) exhibit a complex and transcriptomic response when exposed to thermal environmental stressors. Under current ocean conditions (15 °C), the larvae possess a high range of physiological plasticity, by the modulation of adaptive responses including developmental arrest, energy conserving metabolic reprogramming and neuroprotective responses. The mechanisms, resembling typical dauer-like regulation of other nematodes like *C. elegans*, enable survival for up to 60 days. This extended viability may support sustained transmission potential and their persistence in the marine ecosystem.

In contrast, under extreme marine heatwave-related conditions (28 °C), *A. simplex* promotes a rapid and intense stress response by the activation of pathways related to thermal stress, proteolysis and cellular damage. This rapid response reduced resilience under acute heat stress, resulting in a high decline in larval viability within just a week. Therefore, under marine heatwaves that become more frequent and intense with ongoing climate change, *A. simplex* may reshape its distribution, alter transmission dynamics within hosts, and temporarily constrain its persistence in warmer regions. Understanding the significant impact of episodes of temperature extreme events on *Anisakis* distribution is essential to anticipating future shifts in *Anisakis* spp. endemism, and the subsequent epidemiological risk posed by anisakiasis in global marine environments.

## Data Availability

The datasets generated for this study can be found in the Sequence Read Archive (SRA) http://www.ncbi.nlm.nih.gov/sra under BioProject accession number PRJNA1371940.
