# Supplementary material for: Potential impact of marine heatwaves on the survival and transcriptomic signature of free-living third-stage larvae (L3) of Anisakis simplex (Nematoda: Anisakidae)
Source: Front Vet Sci. 2026 Feb 6;13:1758357. doi: 10.3389/fvets.2026.1758357 (PMC12920175; doi:10.3389/fvets.2026.1758357)
Supplement: Supplementary file 4 [file Data_Sheet_1.docx]

Supplementary Material

# Supplementary Figures

## Supplementary Figures


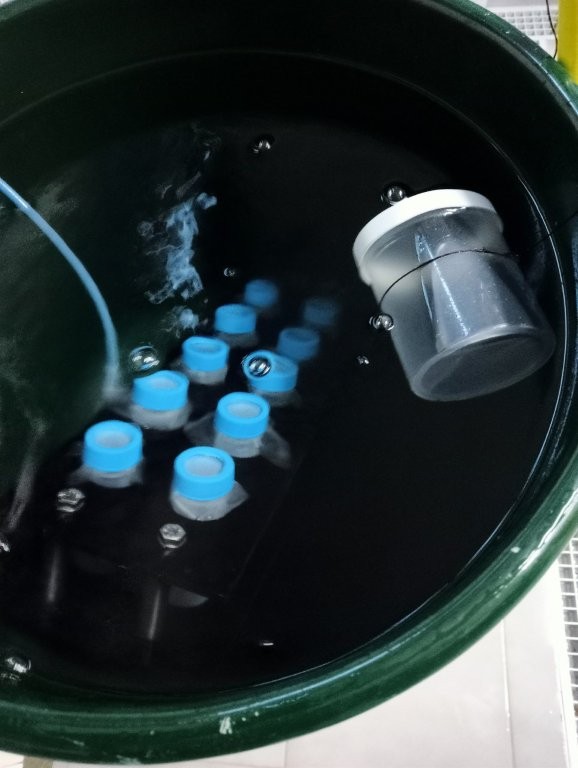


**Supplementary Figure 1.** **Aquarium system used for the survival experiments.**
Image showing the experimental setup used to maintain *Anisakis* larvae under controlled conditions at **15 °C** and **28 °C**.


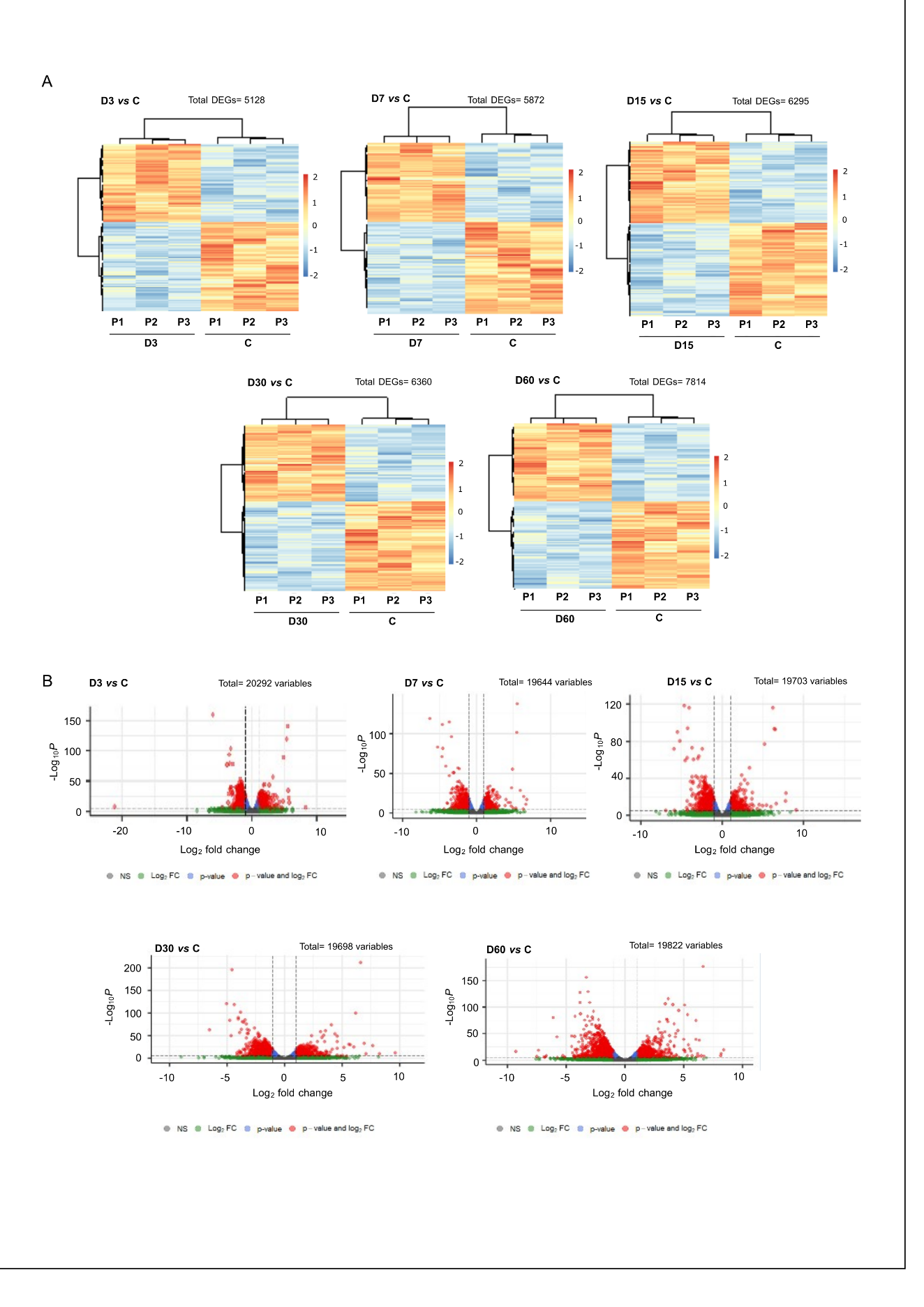


**Supplementary Figure 2.** **Heatmaps and volcano plots illustrating differential gene expression under experimental conditions at 15 °C.** (A) Heatmaps display hierarchical clustering of differentially expressed genes (DEGs) across multiple time points relative to the control (D0). Expression levels are represented by color gradients, with orange denoting upregulation and blue indicating downregulation, thereby revealing distinct transcriptional patterns. DEGs are shown for each pool (P1, P2, P3) under the respective conditions, with data normalized using the regularized log (rlog) transformation.
(B) Volcano plots depict the statistical significance (−log10 *p*-value) and magnitude of expression changes (log_2_fold change) for DEGs. Significantly regulated genes (*p*-adjusted < 0.05) are highlighted in red, whereas non-significant genes are represented in black and green.


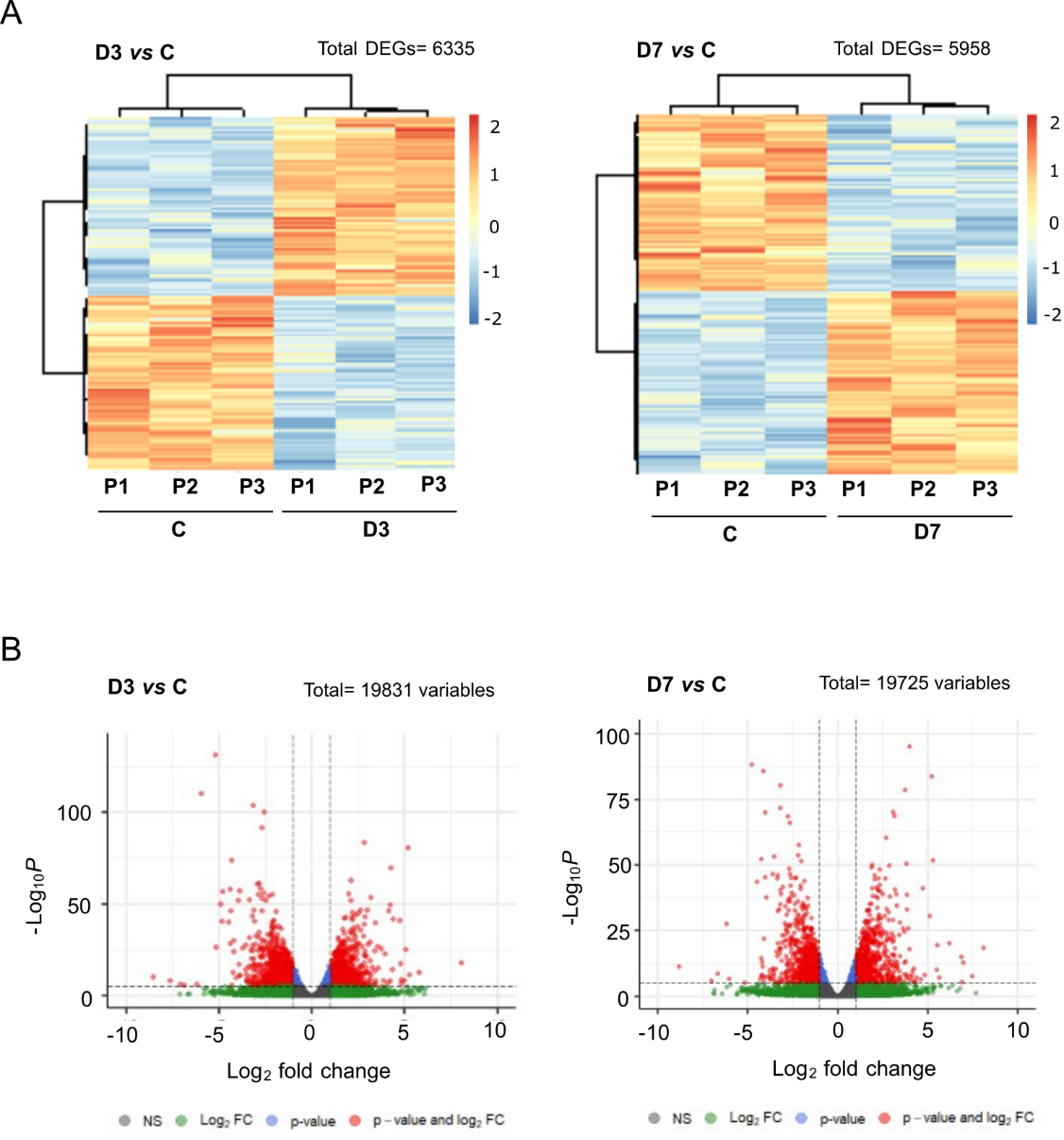


**Supplementary Figure 3.** **Heatmaps and volcano plots illustrating differential gene expression under experimental conditions at 28 °C.** (A) Heatmaps present hierarchical clustering of differentially expressed genes (DEGs) across multiple time points relative to the control (D0). Expression levels are represented by color gradients, with orange denoting upregulation and blue indicating downregulation, thereby revealing distinct transcriptional patterns. Genes overexpressed at a given time point generally exhibit reduced expression in the control, and vice versa. DEGs are shown for each pool (P1, P2, P3) under the respective conditions, with data normalized using the regularized log (rlog) transformation. (B) Volcano plots depict the statistical significance (−log10 p-value) and magnitude of expression changes (log_2_fold change) for DEGs. Significantly regulated genes (p-adjusted < 0.05) are highlighted in red, whereas non-significant genes are represented in black and green.


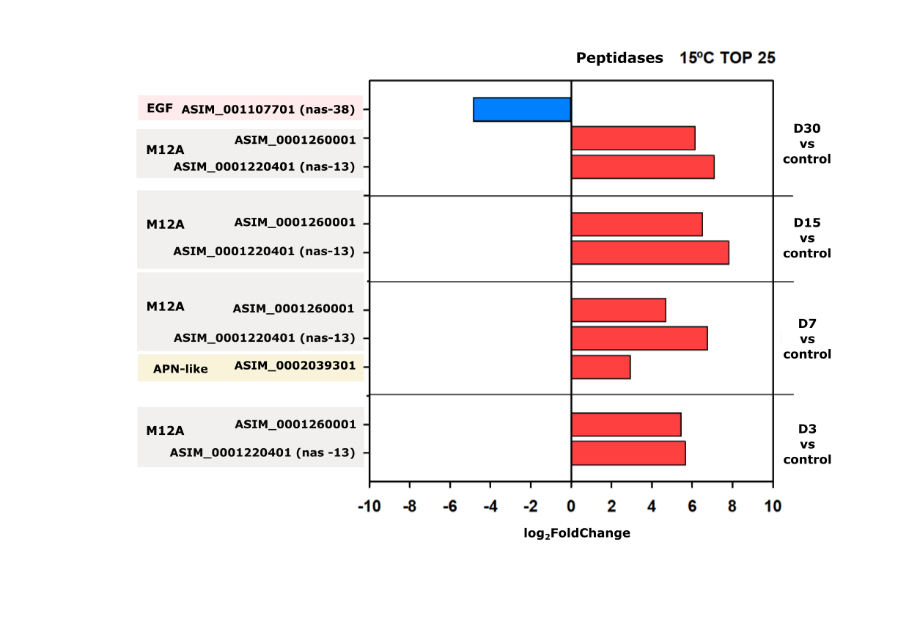


**Supplementary Figure 4.** **Differential expression of peptidase genes at 15 °C.** Bar plot showing the top 25 peptidase genes with the highest absolute log2 fold change under experimental conditions at 15 °C. Each horizontal bar represents the expression change of a gene at a specific time point (D3, D7, D15, or D30) relative to the control (D0). Red bars indicate upregulation, while blue bars denote downregulation. Peptidase family classifications and transcript accession numbers are provided. Orthologous genes in *Caenorhabditis elegans* are shown in brackets. Data reflect temperature-dependent transcriptional responses and were derived from regularized log-transformed counts. EGF: epidermal growth factor-like; M12A: metalloprotease family M12; APN-like: aminopeptidase-like.
